# Folic Acid and Coenzyme Q10 Ameliorate Cognitive Dysfunction in the Rats with Intracerebroventricular Injection of Streptozotocin

**Published:** 2012

**Authors:** Hamid Reza Dehghani Dolatabadi, Parham Reisi, Hojjatallah Alaei, Hamid Azizi Malekabadi, Ali Asghar Pilehvarian

**Affiliations:** 1 *Department of Basic Sciences, Isfahan Payame Noor University, Isfahan, Iran*; 2 *Department of Physiology, School of Medicine, Isfahan University of Medical Sciences, Isfahan, Iran*; 3 *Biosensor Research Centre, Isfahan University of Medical Sciences, Isfahan, Iran*; 4 *Department of Basic Sciences, Islamic Azad University Khorasgan Branch, Isfahan, Iran*

**Keywords:** Alzheimer disease, Coenzyme Q10, Folic acid, Passive avoidance learning, Streptozotocin.

## Abstract

**Objective(s):**

The present study aimed to investigate the effects of a fat soluble antioxidant, coenzyme Q10 (CoQ10) and folic acid on learning and memory in the rats with intracerebroventricular injection of streptozotocin (ICV-STZ), an animal model of sporadic type of Alzheimer's disease.

**Materials and Methods:**

The lesion groups were injected bilaterally with ICV-STZ (1.5 mg/kg b.wt., in normal saline). In the treated groups, rats received folic acid (4 mg/kg; i.p.) or CoQ10 (10 mg/kg; i.p.), either alone or together, for 21 days. Passive avoidance learning test was used for evaluation of learning and memory.

**Results:**

The results showed that learning and memory performance was significantly impaired in the rats with ICV-STZ (*P*< 0.001), however CoQ10 and folic acid, either alone or together, prevented impairments significantly (*P*< 0.001), as there was not any significant difference between these treated lesion groups and control group.

**Conclusion:**

The present results suggest that CoQ10 and folic acid have therapeutic and preventive effects on cognitive impairments in Alzheimer’s disease.

## Materials and Methods

Male Wistar rats (300±20 g; 12 months old; provided by the Pasteur Institute of Iran) were housed four per cage and maintained on a 12 hr light–dark cycle in an air conditioned constant temperature (23±1 °C) room, with food and water made available *ad libitum*. The Ethic Committee for Animal Experiments at Isfahan University approved the study. Animals were divided into five groups (n= 10-11 in each group): the sham, the lesion, the lesion + folic acid, the lesion + Q10 and the lesion + folic acid + Q10.

The rats were anesthetized with chloral hydrates (400 mg/kg, i.p.) and their heads were fixed in a stereotaxic frame. A heating pad was used to maintain body temperature at 36.5±0.5 °C. The skull was exposed and two small holes were drilled and injection canula was lowered into the lateral ventricles (AP=-0.8 mm; ML= ±1.6 mm; DV= -4.2 mm) (30). Injection canula was connected to a Hamilton syringe attached to a micro-injector unit. The lesion groups received a bilateral ICV injection of STZ (1.5 mg/kg, body weight in saline, 4 µl/injection site) as in previous studies (9). The sham groups underwent the same surgical procedures, but same volume of saline was injected instead of STZ.

From the second day after surgery, rats in different treated groups received folic acid (4 mg/kg, in saline; i.p.) or CoQ10 (10 mg/kg in corn oil; i.p.) or both of them for 21 days. Animals in the sham and the lesion groups received same volume of placebo.

After 3 weeks of intracerebroventricular injection of STZ and treatment, the rats were tested with passive avoidance learning (PAL). The apparatus consists of two separate chambers connected through a guillotine door. One chamber was illuminated, while the other was dark. The floor of both chambers consists of steel grids, used to deliver electric shocks. On the acquisition trial, each rat was placed in illuminated chamber while its back was to the guillotine door. After 10 sec of habituation, the guillotine door separating the illuminated and dark chambers was opened. The guillotine door was closed immediately after the rat enters the dark chamber, and an electric foot shock with 1.5 mA intensity was delivered to the floor grids for 3 sec, then the rat was removed from the dark chamber and returned to its home cage. Twenty four hr and one week later, retention latency time to enter the dark chamber was taken in the same way as in the acquisition trial, but foot shock was not delivered, and the latency time was recorded up to a maximum of 300 sec.

Data were analyzed using the SPSS 16 for Windows. The data were analyzed statistically by repeated measures ANOVA followed by Dunnett's Multiple Comparisons test. The significant level was set at *P*< 0.05. Results are expressed as mean±SEM.

## Results

**Figure 1:**
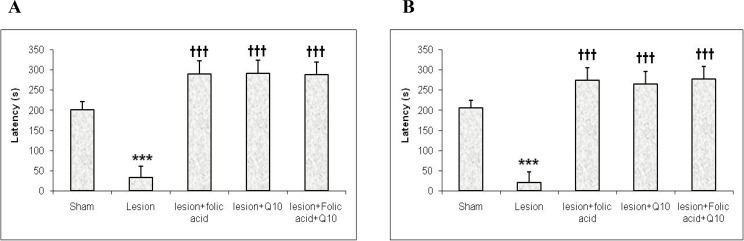
Effects of folic acid and coenzyme Q10 on step-through latency in the rats with intracerebroventricular injection of streptozotocin, 24 hr (A) and 1week (B) after PA acquisition. Data are expressed as mean±SEM (n = 10, 11). *** P< 0.001 with respect to the sham group, †††* P*< 0.001 with respect to the lesion group.

The mean initial latency in the acquisition trial was unchanged among the groups. Results from the retention phase of PAL as measured by mean retention latency time have shown twenty four hr after acquisition phase, mean retention latencies in the lesion group (32.66±27.62 sec) was less than the sham (200.63±20.175 sec; *P*< 0.001), the lesion+folic acid (290.33±31.9 sec; *P*< 0.001), the lesion+Q10 (292±31.9 sec; *P*< 0.001.) and the lesion+folic acid+Q10 (288±31.6 sec; *P*< 0.001) groups (Figure 1A); and one week after acquisition phase, mean retention latencies in the lesion group (20.16±26.82 sec) was less than the sham (205.42±19.58 sec; *P*< 0.001), the lesion+folic acid (274.5±30.97 sec; *P*< 0.001), the lesion+Q10 (264.83±30.97 sec; *P*< 0.001.) and the lesion+folic acid+Q10 (277.16±30.97 sec; *P*< 0.001) groups (Figure 1B). However, the lesion+folic acid, the lesion+Q10 and the lesion+folic acid+Q10 groups comparing to the sham group didn’t have any significant difference.

## Discussion

The results showed that folic acid and CoQ10, either alone or together prevent learning and memory decline in rats with intracerebroventricular injection of STZ; nevertheless simultaneous application of these two substances did not have better effect than their single application.

Cognitive deficits and biochemical and structural changes in the brain of rats with ICV-STZ mainly were attributed to generating free radicals and altering glucose energy metabolism by depleting ATP synthesis (6, 9, 31).

Studies have demonstrated that the normal cellular energy metabolism is necessary for normal functioning of the brain (28), and when availability of ATP is low in the brain, faulty amyloid precursors protein (APP) metabolism and hyperphosphorylation of the tau-protein are high, that induce production of neuritic placques and neurofibrillary tangles, respectively, which are prominent histopathological markers of AD (28). It has been demonstrated that intracerebroventricular injection of STZ causes impairment of neural glucose metabolism leading to reduction of ATP and creatine phosphate formation (32, 33), but it was seen that CoQ10 can restore this impaired glucose energy metabolism effectively in ICV-STZ rats (9).

In addition, it is revealed that through improvement of glucose energy metabolism and production of acetyl CoA, and protection of choline acetyltransferase (ChAT) activity, CoQ10 protects cholinergic neurons in the brain of ICV-STZ infused rats that cholinergic neurons are damaged severely (9,34,35).

Oxidative stress plays a pivotal role in Alzheimer’s (36). Oxidative stress damages neuronal membranes lipids and proteins, through generation of free radicals, and therefore damages membrane integrity (37) and reduces the number of nerve cells (38). Because both CoQ10 and folic acid are powerful antioxidant and they have free radical scavenging property, they can reverse the free radical induced damages seen in neurodegenerative diseases and resultant learning and memory defects (23, 39, 40), as seen in our results.

Studies have shown reduction of folic acid as seen in AD results in hyperhomocysteinemia (20, 26, 41, 42). Folic acid deficiency and hyperhomocysteinemia impact neurons by affecting antioxidant defense systems and impairing DNA repair that induces neural cell apoptosis (43, 44). Folic acid supplementation by converting homocysteine into cysteine can increase level of reduced glutathione in all the regions of brain (45). Also, this has beneficial effects in increasing the superoxide dismutase and catalase activities that are protective enzymes against highly reactive free radicals in the brain (46, 47).

Because folic acid and CoQ10 are both reduced in AD patients (26, 27), and folic acid can potentiate endogenous synthesis of CoQ10 (17, 18), therefore, usage of folic acid probably aids to improve AD by increase CoQ10. Hence, administration of folic acid and CoQ10 in AD apparently have same effects, and our results verifies it, because co-administration of CoQ10 and folic acid had same effects on learning and memory as there were in separate administration of them.

Finally, similar to our protocol, other studies have started their intervention, one or two days after ICV-STZ for a period of time (48, 49); and most of them believe that positive effects of their interventions were due to amelioration of ICV-STZ complications, rather than reduction of STZ effectiveness directly; however we do not reject this possibility.

## Conclusion

In conclusion, our findings suggest that CoQ10 and folic acid, either alone or together, protect learning and memory performance in the rats with intracerebroventricular injection of streptozotocin. The data correspond to the possibility that prophylactic treatment with CoQ10 or folic acid can offer protection against Alzheimer’s disease.
